# How migrants keep Italian families Italian: *badanti* and the private care of older people

**DOI:** 10.1108/IJMHSC-08-2015-0027

**Published:** 2017-06-12

**Authors:** Silvana Rugolotto, Alice Larotonda, Sjaak van der Geest

**Affiliations:** 1 International Committee of the Red Cross, Verona, Italy; 2 Department of Anthropology, Brown University, Providence, Rhode Island, USA; 3 Department of Medical Anthropology, University of Amsterdam, Amsterdam, The Netherlands

**Keywords:** Italy, Migrants, Women, Older people, *Badanti*, Care giving

## Abstract

**Purpose:**

The purpose of this paper is to describe how migration affects the care of older people in Italy.

**Design/methodology/approach:**

The paper is based on anthropological fieldwork by one of the authors. This consisted of in-depth interviews with 20 “*badanti*” (migrant caregivers), with relatives of older people and with social workers in the city of Verona, Italy. It further included extensive study of secondary materials on the topic of migrant care of older people.

**Findings:**

*Badanti*, Italian families and older people find themselves locked in an uneasy contract: *badanti* because they are exploited and often unable to find better, formal employment; Italian families because they are aware that they fail to render their moral duty to their aged parents and grandparents; and older people because they feel neglected and maltreated by their children. Yet the three parties also rely on each other to make the best of a precarious situation. The relationship between *badanti* and Italian elderly highlights the contradictions within Italian politics on care and migration. This case study shows how migrants help Italian families to hold on to the tradition of family care for ageing parents.

**Research limitations/implications:**

The small sample of *badanti* and families provides a detailed and profound insight of the complexity of elder care in Italy but does not allow generalisation for developments in the country as a whole.

**Practical implications:**

Policy makers should take notice of the indispensability of informal migrant care in present day Italy.

**Originality/value:**

The originality of the paper lies in the in-depth conversations with *badanti* and in the way in which elderly care is contextualised in the Italian tradition of care and present day politics.

## Introduction

Two demographic processes deeply affect the changes occurring in the world today and Europe in particular: ageing and migration. This paper addresses their intertwinement. The rapid ageing of society leads to various imbalances, namely economic burden (how to pay for an ever growing “unproductive” population), shortage of care (how to manage adequate care for dependent older citizens) and relational stress (how to give sufficient attention to older parents and grandparents).

The economic burden is mainly a political issue. Politicians and economists in European countries reassure the electorate that there is no reason for panic. They claim that they will be able to pay old people’s pensions and provide care for the coming years, provided people are willing to work a few years longer than the generation before them. In Italy, however, it has become clear that the system is close to collapse. Most older people are unable to fully support themselves with their small pensions, especially when the need for part- or full-time care arises. Adult children, facing increased taxation and loss of working benefits in the current circumstances of economic crisis, often struggle to help their elderly parents. In addition, they must often support the younger generation who are confronted with job insecurity or outright unemployment[1].

The shortage of care and subsequent relational stress affect many people’s personal lives. The inability to spend more time with the older generation, to give them support and, when needed, practical care, has become a moral dilemma for many. Loneliness is one of the main complaints of older people in Western Europe. Admitting older people to nursing homes when they become more dependent may solve practical problems in some countries, but it does not always reduce the feelings of unease and guilt among the younger generation. In Italy, where sentiments of familism are strong, the failure to provide personal care for older relatives brings shame to the family. This disapproval of lacking family care is underscored by state ideology[2].

In addition to a rapidly ageing population, a second demographic phenomenon transforming Western Europe is the immigration by people from poorer countries. Labour migration to Western Europe started in earnest around 1960, but had been taking place for several decades prior. This migration to Europe and the greying of the continent are interlinked. One of the largest niches for migrant labour in Europe is the care sector (Ungerson, 2004; Van der Geest *et al.*, 2004; Lutz, 2008, 2011; Slany *et al.*, 2010; Triandafyllidou and Marchetti, 2013; De la Cuesta-Benjumea and Roe, 2014; Marchetti, 2015). Destremau (2007), in an overview of studies about domestic work and migration, spoke of an ethnicisation and informalisation of this type of work. Näre (2012), who did her research in Naples, views migrant domestic workers from the perspective of ethnification and racism.

Our exploration of elderly care given by migrant women in a European country is a response to Parreñas (2012) call for research into the different influences of globalisation and migration on care (see also Razavi and Staab, 2010). A central concept in the burgeoning number of studies in this field is the “global care chain” (Hochschild, 2000; Cheng, 2004; Parreñas, 2012; Yeates, 2012). This concept refers to the migratory processes of women who leave their own families to provide care in other families in a more profitable setting; in the homes these women leave behind, they are replaced by other women with similar intentions, and thus a chain forms. Researchers studying these chains of care focus among other things on racism, gender inequality and exploitation (Chang, 2000; Cheng, 2004; Yeates, 2012; Christensen and Guldvik, 2013). Razavi and Staab (2010), Yeates (2012) and Walton-Roberts (2012), however, argue that a “chain” is too linear a metaphor, since both the work and the migration routes of carers are complex. In-depth ethnography, which forms the basis of this paper, is able to point to the erratic nature of the micro-politics that underlie the position of female migrant caregivers.

## Italy

The demographic and economic situation in Italy is particularly poignant. In 2000, 18 per cent of the country’s population was above 65 and the expectation is that by 2020 that percentage will be 24 per cent. With the growing number of women looking for salaried work outside the home, the need to hire someone to care for older relatives is increasing (Scrinzi, 2008; Da Roit and Naldini, 2010; D’Onofrio, 2011). Castagnone *et al.* (2013, p. 15) estimate that between 1994 and 2011, the demand for home caregivers quadrupled. This need has been met with an exponential increase in migrant worker employment (+92.6 per cent from 1994 to 2011), such that foreign domestic workers have come to be considered a “gift from heaven”. The official number of migrant domestic workers in Italy in 2012 was 807,000 (INPS, 2012).

When they arrive in Southern and Central Europe, migrants often work in private houses. Statistics are difficult to obtain and unreliable since much of the work takes place informally and/or illegally. Da Roit (2007, p. 266) estimates on the basis of several studies that in 2006 about 800,000 migrant women cared for older people in Italian families. She quotes Pollastri and Tozzi (1999), who calculated that in the late 1990s, 10 per cent of Italian households with a person over 65 – about 750,000 families – were hiring a worker; their calculation was based on a national survey (see also Polverini and Lamura, 2004). Informal employment of a migrant caregiver seems mainly an urban phenomenon, but has also been reported in rural areas. Approaching people who are involved in the business of private informal and/or illegal care is difficult for reasons related to their illegal status, as will be elucidated below. This paper is an attempt to sketch and elucidate the complex picture of migration and informal care for older people in Italy. We draw attention to the somewhat hidden transactions of the two main players: the Italian middle generation and the migrants.

## Fieldwork

Migrant caregivers in Italy and elsewhere have become a popular topic for researchers and we have by now an impressive body of literature about them, but relatively few of these publications are based on anthropological ethnographic fieldwork. The views and experiences of the three parties involved (employers, the elderly and migrant women) are scarcely present in the literature, and direct observations of the work situation of these migrant women nearly non-existent. One exception is Da Roit and Naldini (2010), who interviewed employers of migrant caregivers in Milan. Another is Näre (2012), who carried out participant observation among five migrant women in Naples and interviewed 89 migrant women, employers and other relevant persons.

The first fieldwork for this study was carried out ten years ago in Verona, from mid-May to mid-July 2005 (Rugolotto, 2005). Verona, with a population of nearly 250,000, is an important area for the manufacture of textiles, machinery, shoes and processed food. Architectural treasures such as the Arena, a Roman amphitheatre (First Century AD) and various churches plus Shakespeare’s Verona-based love story of the ill-fated Romeo and Juliet have made the town a flourishing and permanent tourist attraction. Most Veronese citizens enjoyed a high standard of living at the time of the research.

The number of older people in Verona is very high: the ratio between those over 65 years and children under six years is 4 to 1, and those over 65 years represent 20 per cent of all Veronese citizens. Among these older people, 11 per cent are said to be disabled and in need of care, while only 3 per cent live in nursing homes. The remaining 97 per cent of elderly rely on the traditional culture of family responsibility (named “family welfare” by politicians). The government provides a “care allowance” to families to support home care. However, many families have to rely on external helpers in order to be able to provide care for their older people. It has been estimated that, at the time of fieldwork, in the Veneto Region (where Verona is situated), with a population of 850,000, about 21,000 immigrants provided home care to frail elderly (Fabris, 2004).

The migrant women who carry out this care work are popularly called *badanti*, which literally means “caregivers”, but the term has become somewhat derogatory and discriminatory as it is only applied to migrant women. *Assistente familiare* (care assistant) would be a more correct term (Näre, 2012, p. 23). Another reason for the negative connotations of the term is that many older people resent the presence of *badanti* and criticise their children for hiring them. *Badanti* typically work in domestic settings. When they are formally employed in a care institution – which does increasingly occur – they are not called by the term. In spite of the somewhat demeaning sound of *badante* (singular of *badanti*), we will nevertheless use it in this paper as it is commonly used “in the field”.

Initiating contact with *badanti* proved difficult. The *badanti* were reluctant to meet a researcher; many of them had an insecure position and tried to avoid publicity. Families who employed *badanti* also preferred to remain unknown. They did not want to lose their *badanti* and tried to avoid criticism for not personally looking after their older relatives. Despite these obstacles, the researcher (SR) managed to meet and speak with 20 immigrant caregivers and 5 relatives of older people. Obviously, 20 *badanti* constitute a very small sample for readers interested in quantitative data, but the detailed information that some of the women provided, in combination with more casual observations by the first two authors, have resulted in a true-to-life description of the daily – more hidden – aspects of this informal contract between migrants and Italian families.

Interestingly, it proved impossible to meet any older people in their houses[3]. Both the relatives and the *badanti* were extremely reluctant to allow the researcher into the house. We suspect that the relatives wanted to prevent the elderly from complaining to an outsider about their delicate situation; the *badanti* simply had no right to allow (other) strangers into the house. The best place to approach the *badanti* was, therefore, not in their workplace but at the centre of a charitable association.

Six *badanti* provided substantial information about their situation. They recurrently and spontaneously raised many specific themes. Some repeatedly told the researcher how nice Italian *vecchietti* (sweet diminutive of older people) were. Obviously, *badanti* applying for a job present themselves as patient and caring persons, able to look after sick and older people. The researcher was conscious of the possible difference between rhetoric and genuine sentiments. Excerpts from three somewhat arbitrarily selected (most informative) conversations are presented here to sketch how *badanti* view the situation in which they find themselves.

The fact that fieldwork for this study took place a decade ago raises the question of how relevant these observations are today. We will briefly return to this question in our concluding discussion. For the moment, suffice it to say that the central intuition of this paper, the paradox of migrants rescuing the Italian moral economy, has been largely overlooked in the existing studies on the phenomenon. Furthermore, the diachronic character of this paper allows us to reflect on what has changed over the past years in the social and cultural position of *badanti* in Italian society.

## Conversations with three *badanti*

### A Moldavian *badante*

One interview was with a 27-year-old Moldavian woman who had left her country four years ago in search of work and a better future. Before she left Moldavia she had been a law student, but she left university, endured the abuses of immigrant trafficking and paid the fees of passage to arrive in Italy. In Verona, she contacted a Moldavian immigrant already working as a *badante*, who introduced her into the hidden job network:

It was my first job after three months. I had to care for an older couple living in their daughter’s house in a nearby town. The daughter worked outside the home. She gave me instructions about the care. It was a heavy, full-time job. I still had problems with the language. I had no contacts with the outside world and had no working contract […] It was like a tomb. I could not breathe […] I noticed that the old lady often argued with the daughter […] also I found that the neighbours came to the house, not to visit the old couple but to spy on my activities, probably to report to the daughter. It was a short job; the couple moved to stay with another daughter. They no longer needed my services. We never met again.

In this excerpt, some themes already emerge: the illegal immigrant/illegal job, isolation felt by the *badante*, disagreements between older people and their children (daughters in particular) and neighbours checking on the behaviour of the *badante*:

After that I got another job for eight months, to assist an old lady of 92. Still full-time […] no time to study Italian. But I could watch television and learn a little. The daughter was the person in charge. She removed all the valuable things from the apartment before my arrival. But I had a good relationship with the old lady. She pretended to have small secrets with me, such as to go to shops to buy something. Then they dismissed me because they did not need me anymore.

After that I got my present job. Always through our network [there are many Moldavian caregivers in Verona], I had been asked to present myself to a family in the outskirts of Verona. You know, now I reside officially with another Moldavian caregiver. This happened two years ago. The older couple, living alone, needed a maid to do the hard work and shopping and to accompany them to the doctor. It was a full-time residential job, but with regular days off. The two were in good health […] You must know that we badanti are asked to do everything, nursing also. Some Moldavian caregivers give injections, change dressings, etc.

From the beginning, this family wanted to register me with a regular contract and insurance, in case I needed medical care and also to avoid problems with the immigration office. […] Last December, the old lady fell sick and died in the hospital. At the same time the old man broke a leg. Due to that, with the old lady at the hospital and also caring for the immobilised old man at home, the family decided to employ a nurse to carry out the medical work. It was a fair solution. Soon the old lady died. After recovering, the old man wanted to keep me as badante but only during daytime. He thought that as a girl I should not stay there [in the apartment with him] during the night. He could call his son, who was living nearby, if something happened. This old man is a good person. I do everything for him. I want to be sure that before leaving, at the end of the day, everything is in order. There is a lot of respect between us. All the families should be like this. I have even time to do a part-time job if there is the opportunity.

That is respect. Respect is what is lacking in Italian families toward us immigrant caregivers. I told you already: family members that come to check my belongings in my absence! It is so offensive. We are human beings caring for their old parents and they check our belongings! It is painful. You know, it is not just me; also the other caregivers have to pass through the same checks. These people consider their money, jewellery and paintings more valuable than their elders!

### A Romanian *badante*

Another *badante* was a 37-year-old Romanian woman, who was divorced and had been living in Italy for the past six years. When she arrived in Italy, she first went to Rome where her sister was living. She then moved from one city to another, for various short-term assistance jobs that she learned of through the immigrants’ network. She had worked as a *badante* both in families and in nursing homes:

My first job was to replace a Polish badante during her annual leave period. It was a 24-hour a day job. I had to sleep in the same room as the old lady, to care for her and prevent her from falling out of bed. Her son gave me all the instructions, including the type of food to cook. I could never cook my own food. I remember that there were arguments between the lady and the son, because, as I understood, the apartment belonging to the lady had been sold and the lady was always reminding him of that offence [saying they could at least have waited until she was dead]. After that short service and because I was an illegal worker, I could benefit from a certain law to get a working visa. In Italy there is a contradiction, because we have to be illegal or clandestine to be able to become legalised. The system forced us to be illegal to apply for regularisation! That is wrong! Anyway, after that I was able to get a job in an old people’s nursing home. The older people there were complaining that they wanted to be in their own home and that they wanted the children to come to the nursing home to keep them company. But if they stayed at home, they would need at least two badanti because of their disabilities. In the Italian system, to look after an old parent and to have one or two badanti is too big a responsibility for the children. My parents are 68 and 71 years old. My younger son stays with them; I send money to maintain the whole family. My other son, 15 years old, is now with me in Verona, but illegally. I could not show to immigration officers that I have a place to stay. Unfortunately, my parents in Romania expect too much from my salary [which is ten times their own pension], but they do not know how expensive life here is. We have arguments about how much money I can save here.

This woman was upset about the complaints of the older people against their children who had placed them in nursing homes. She further remarked that Italian laws create more illegal immigrants than they claim to stop. Case in point: this mother kept her younger son with her clandestinely because it was the only way that she could care for him.

### Another Moldavian *badante*

A third *badante* who was interviewed at length was a 42-year-old Moldavian woman. She spoke Italian well and considered herself a real *badante*. She had left her country three years ago when she realised that with her disabled husband (from wounds sustained during the war) she had to earn money to support the family. She got the idea to become a *badante* from her sister, who was already working in Italy. She started to study Italian and cooking at home in order to be ready when her sister found a job for her. With her new job, she can pay for her husband’s medical care. Furthermore, she has been able to begin rebuilding their house that was destroyed during the war. She knows that her job as a *badante* is full time but temporary, and depends on the length of the lives of her clients. That suits her, because between one job and another she goes to visit her family in Moldavia. In Verona, she has a small flat so that her daughter and husband can also come and live there. She said: “It was important because she [the daughter] was pregnant and I wanted her to receive good maternity and childcare; in Italy, this care is provided free, also for illegal workers”. This woman seemed to be much in demand by Italian families. At the end of the interview she received a telephone call for a new assignment:

Maybe everything has gone well with me because when I arrived in Italy I had already had all the horrible experience of the war. So, I am very caring about people and things. I remember the first client was a widower; his wife had died just one week before my arrival. I arrived with a tourist visa to take the job immediately. I was trained as a nurse although I did not complete the course, but I feel comfortable giving assistance. Anyway, the old man was not sick but felt very lonely. He used to call me by the name of his beloved wife. I let him do so. His children soon began to trust me and gave me responsibility for domestic affairs. I got on well with all of them. Also with the money I was free. At the beginning, the son explained to me how to estimate the domestic expenses, after that I did it by myself. Because the family income was below what is required by the Italian Labour law, they could not give me a registered contract, but it was good for me to stay as an irregular worker. I was treated as an older sister. After one year the old man was hospitalised and after one week he died. I attended his funeral. I found the ceremony very nice, elegant, with flowers and songs. In my country, because of lack of money we cannot do that. After that I did other assistance, assigned by this charitable association. The last job was temporary assistance to two persons with mental problems; mental problems cannot be cared for at home, too difficult. They had to wait until there was a place free in a nursing home. They were placed there last week, so now I am here available for another client. I am already on the waiting list. I have many good references from the previous jobs. The operators here have contacts with the families where I worked which serve as oral recommendations.

The three *badanti* quoted above all have in common the fact that they are women who travelled from a low-income land to Italy to earn money to send home. Their stories also overlap in the sense that they took over the task of sons, daughters or daughters-in-law who were unable or unwilling to take care of their older relatives. Furthermore, all said that they had access to Italian medical services. But the experiences of the *badanti* also differed. Some felt that they were not accepted by the older people and mistrusted by their younger relatives. Others succeeded in building up a good relationship with the Italian families. Some said that they were exploited; others liked the work they were doing. Some worked illegally, others managed to get a work permit. That same diversity appeared in conversations with members of the Italian families.

## Conversations with family members

### The experience of a son

I am a man, 55 years old, but because I am single my mother who is a widow expects me to care for her as if I were a daughter. Since about two or three years she needs help both for domestic work and for her personal hygiene. When I introduced a badante to break that vicious circle of moral blackmail it became a tragedy. My mother theatrically demonstrated how bad a son I was to leave her alone with a foreign badante. Nevertheless, I did bring a badante to her house. A young immigrant, 30 years old, from the Ukraine, has been working there for six months. However, my mother still refuses to be helped in her personal hygiene and never leaves the badante alone, afraid that the badante will steal or discover I do not know what. My mother takes her meals alone because, as she complains to me, she cannot eat without teeth in front of a foreign person! My mother considers this young woman a guest and refuses to pay her. I pay her salary secretly. This worker is illegal, it has been agreed that it is convenient for her and for us too. At the moment she is looking for another job. It is a temporary solution. With a mother who says that caring for her is not the duty of a foreign person but a moral obligation for her children, I do not know how this will end.

### The views of a daughter

I have only indirect experience with a badante. My brother, who lives with our old parents, employed a forty-year-old Romanian woman about two years ago. She is the mother of two children and divorced. She is an illegal worker and travels back and forth to Romania with a tourist visa. I don’t know how it happened, but after a few months my brother and the badante became lovers and the woman started to give orders to the others, including my parents. She is now in charge of the money of the house, and while I was there she used to sleep until ten in the morning. So in my opinion, they [badanti] are just women coming to Italy to make money and find some idiot men to marry. I have a friend who also fell into the same trap. After a few months this man, a teacher, found himself married with the badante and his mother placed in a nursing home because she was disturbing the household! These women are very clever in using the emotions of a family member to get themselves comfortably settled.

### Views of another daughter

Since I am the daughter and single, fifty years old, my parents and all the family members have no doubt that it is my moral duty to care for my parents. All these years I have been working and doing all the domestic work at home and serving my parents. Recently their health deteriorated a lot. Both of them are eighty years old, so now I have to take care of them. They never thought that it would be unfair to use me as their maid. When I suggested employing a badante they replied that it was a public shame and a sign of my lack of love to propose that. We could afford to pay a badante but they said that what should be done by a daughter does not need to be paid for. Now the situation has become unbearable, emotionally and physically. I cannot cope anymore. I do not know how to get out of this.

## Discussion

In the introduction to her book on migration and domestic work in Europe, Lutz (2008) points at three crucial differences between domestic work and other transnational work arrangements. First, domestic work cannot be outsourced to low-salary countries, as can call centre employment and industrial production. Foreign caregivers can only carry out their work in European households[4]. Second, this implies that these migrants must integrate into and adapt to the intimate sphere of local families. This requires social skills, as they have to take up tasks at the core of what for them is a new culture. Finally, this situation leads to emotionally confusing and ambiguous situations. Although they take over the tasks of adult children (in caring for parents), and also of parents (in caring for children), they are not meant to replace children or parents. Relatives must, on the one hand, rely on the good care of these hired workers, but are, on the other, afraid that the immigrants’ care will become “too good” and will marginalise them in their own home. Employers and employees are thus caught in an awkward contradiction.

There were many examples of this mutual discomfort in the conversations with *badanti* and relatives. The former complained that they were expected to carry out highly intimate activities but were still considered with suspicion and treated as strangers. The latter expressed worries that these intimate strangers may take advantage of their position to steal both material and emotional “property”. The fear that a *badante* will establish a relationship with either an older man whom she looks after or with a younger male relative is particularly widespread.

At the same time, however, *badanti* and relatives of older people are aware that they need one another and are willing to make compromises. The arrival of legal and illegal immigrants who provide care for older Italians may be criticised by older people, relatives, political authorities and even by the *badanti* themselves, but all four parties are mainly satisfied with this new phenomenon. Our exploration suggests that public complaints about *badanti* mostly mask private approval (cf. Razavi and Staab, 2010, p. 420).

### Badanti

Working as a *badante* gives migrant women a solution to their problems at home. Their Italian salaries enable them to help their own relatives, even though this means that *badanti* themselves may have to live on extremely low resources in order to be able to send remittances home. Salary ranges still appear low in comparison to the Italian cost of life, especially in northern Italy (despite full-time employment often reaching 54 hours per week for live-in caregivers). This establishes a difference between *badanti* who send remittances and those who have brought their family along.

The *badanti*’s position is vulnerable, as for most their employment status is strictly connected to the legitimacy of their stay on Italian territory (Castagnone *et al.*, 2013)[5]. Since 2009, Italian legislation considers illegal stay in the country a criminal offence (Law No. 94/2009) (although more recently action was undertaken to decriminalise this). Many workers have therefore become less available for irregular work since 2009, although estimates suggest that almost 50 per cent of caregivers still work in full or partial irregular employment (Castagnone *et al.*, 2013, p. 31). There is a great demand for *badanti* and no one really wants to prevent them from doing what they are doing. An abrupt end to a job due to the death of an older person usually does not pose a threat because of this high demand, although there are exceptions (Fedyuk, 2009).

Undoubtedly, *badanti* are exploited, but it would be a simplification to view them as helpless victims of an unfair system. All of them demonstrated strong agency in spite of the fact that they had little room to manoeuvre. Many were able to turn their illegal situation into an advantage. It gave them more freedom to move around. Moreover – in contrast to what one would expect – they believed that their illegal status gave them negotiating power *vis-à-vis* the families who employed them. Furthermore, many *badanti* also managed to become legal residents.

Several authors (Parreñas, 2008; Degiuli, 2007; Scrinzi, 2008) have pointed at racism and emphasised that *badanti* are excluded from social and medical services, but the *badanti* in Verona did not complain of such exclusion. They did, however, mention ostracism and everyday discrimination. Colombo and Decimo (2009) distinguish three main forms of relationship between *badanti* and their employers: relations based on “service”, where the relationship is principally based on contractual agreements in which expected services and retributions are established; relations characterised by intimacy, trust and affection, where the *badante* becomes the recipient of family-like attentions; and relations based on subservience and power, in which *badanti* are subjected to the constant threat of dismissal. Similarly, Marchetti (2009) argues that these relationships are characterised by fundamental ambivalence, as the asymmetrical work relationship is accompanied by intense emotional attachment.

Some *badanti* complained about a lack of respect and mistrust, but at the same time they looked down upon the family that could not even look after its own aged parents. Other *badanti*, however, reported very positive experiences and warm relationships with the older people and the younger family members. Mutual respect and gratitude between *badanti* and relatives have also been mentioned in several studies. Fedyuk (2009) describes how the death of an elderly person often distresses the *badante* who cared for him/her. Marchetti (2016), who studied Filipina domestic workers in Rome, mentioned that Roman employers were aware of the hardship of the Filipinas who had left their own families and showed their gratitude by treating them with respect and sometimes even special regard. Some offered help when the Filipinas were in trouble because of their uncertain status. Home care workers may become “fictive kin” as has also been observed for non-migrant caregivers (cf. Karner, 1998; Piercy, 2000).

Stories about love affairs between *badanti* and both the younger and older generations abound throughout Italy. Giancristofaro (2007) discusses the common belief (and fear) that *badanti* manage to convince older Italian men to marry them, consume their pension and then upon the death of their new husband run away with the entire inheritance. Similar stories were also told by relatives in Verona. Giancristofaro, however, admits that she never came across an actual case. These stories indicate that the Italian public recognises, and probably overestimates, the *badanti*’s agency.

The fact that domestic work often implies longer working hours than is officially agreed upon is well known (cf. Aronson and Neysmith, 1996; Karner, 1998; Piercy, 2000). This extension of home care is particularly prominent in the case of migrant caregivers. Degiuli (2007), writing about migrant women providing care to older people in Turin, speaks of “a job without boundaries”, describing how a day and night is filled for a *badante*. She tells of one *badante* who provided 24-hour care as being “locked into a claustrophobic relationship that is hard to break out [of]” (p. 205). It is this locked-up situation, she continues, that renders this occupation nearly invisible to wider society.

The fact that this 24-hour service is a heavy burden was also mentioned by the Verona *badanti*, but it had its advantages as well: these *badanti* had comfortable and cheap accommodation and could thus save more money to send home.

### Older people

Older people were not interviewed in this research. The scant information that we have derives from what *badanti* and relatives told us. Older people seemed divided over the growing role of *badanti*. Some bitterly rejected the “solution” of hiring a *badante* and accused their children of betraying them and failing in their filial duties. Others seemed content with the presence of a *badante*, and felt that having a *badante* was much better and less shameful than being placed in a nursing home.

### Relatives

For relatives who are unable to look after their ageing parents, *badanti* are indeed a gift from heaven. They can now escape the public shame of a nursing institution and avoid the problem of having to combine the personal responsibilities of work and their own nuclear family with the daily care of a dependent older parent. Da Roit and Naldini (2010) found that paid employment for children of dependent older parents is not so much an obstacle to care giving but rather a buffer: they use “employment as a way to reduce the demands and to protect themselves from overwhelming care responsibilities” (p. 548). *Badanti* make the use of that buffer possible.

Marchetti (2015), who interviewed 14 people who had hired *badanti* to care for their older relatives, emphasises the ambiguity of the relationship between employer and employee. Some of the interviewees saw *badanti* as a relief who saved them from physical exhaustion and emotional collapse. Others complained that *badanti* were money minded and did not really care; they mistrusted them and kept close checks on them (see also Triandafyllidou and Marchetti, 2015).

The financial arrangements between families and *badanti* are not always clear. A legal *badante* should earn between 900 and 1,000 euros per month, but irregular employment represents a considerable saving for families who do not have to pay taxes or grant benefits such as maternity leave, sick leave and vacation. Some years ago, hiring a *badante* for one’s older parent(s) was not a privilege exclusively available to a rich upper class. Da Roit (2010, p. 93) writes that middle class and working class households could also employ a *badante* in the grey sector. The costs were between 800 and 900 euros a month (plus food and accommodation). Families with an average income and even the 50 per cent of older Veronese who enjoyed a care allowance of about 400 euros per month could thus afford a *badante*. This may have changed, however, due to the economic crisis.

Finally, *badanti* can also have other ambitions, as the daughter above pointed out when referring to her brother’s affair. As mentioned before, relatives are worried that in addition to the formal payment, *badanti* may acquire additional income by stealing or establishing a relationship with one of the male family members. The romantic and/or scandalous nature of such an affair makes this a popular rumour about *badanti*, but its actual prevalence is difficult to verify (cf. Giancristofaro, 2007).

### The state

The strict European and national rules against migrants are applied flexibly in Italy. The legal twilight in which many *badanti* must operate has advantages for Italian families and the state itself. It gives them – somewhat paradoxically – greater control over *badanti. Badanti* help the Italian state to save on its budget for senior citizens by allowing older people to stay out of nursing homes, and at the same time assist families to provide culturally and morally acceptable care to their older relatives (cf. Da Roit, 2010; Da Roit and Sabatinelli, 2013). “Badanti give a service that is not available within public healthcare, as it is considered too intensive for families and too expensive if acquired through normal services available on the market” (Da Roit and Facchini, 2010, p. 25)[6]. This system of domestic care by migrant women may not be actively sought by the state, according to Marchetti (2013, p. 361), but the state’s active condoning of it might as well be regarded a silent policy. In short, the articulation of legal and illegal circuits maintains the Italian family system in spite of changing work patterns and demography.

It seems unlikely, however, that this “cheap luxury” will last forever for Italian families. Political and economic developments will affect this malleable market. The possibility for Romanian and Bulgarian migrants, who are now EU members, to work legally, for example, could make it easier to employ these migrants legally, but at the same time might deprive the market of cheap irregular labour. This may lead to an increase in the employment of migrants of other nationalities (from Africa, Asia, Latin America), who may be available for lower salaries. Alternatively, given the present economic situation, the government might have to consider whether it should start thinking more seriously about expanding nursing home possibilities, even though they are a despised institution in Italy. Castagnone *et al.* (2013, pp. 56-7) argue that what looks like a “win-win-win” solution (for migrants who easily find employment and legalise their status, for Italian families who are able to afford cheap private care, and for the state which is able to limit its spending on older citizens) is bound to collapse with the growth in the ageing population and due to the fact that regular migrants have access to the welfare system. The solution to this predicament thus seems to depend on government intervention, especially in the face of the present day economic crisis. It seems that the state still bets on the present system of care by *badanti*; in 2009, it offered amnesty to 300,000 illegal migrant domestic workers to prevent Italian families from collapsing[7]. “For policy makers and those responsible for social policies, this system reduces significantly the pressure of the increasing demand for care on social services, and makes the necessity of a substantial revision of public policy in this sector less urgent” (Da Roit and Facchini, 2010, p. 25) (see footnote 6).

## Conclusion

When we compare the observations of ten years ago with those of today, the most prominent development is the growing acceptance of *badanti* as caretakers of both older people and children[8]. To have a *badanti* staying in the house is now a “normal” thing, nothing to feel ashamed or uncomfortable about. *Badanti*, on their part, also feel more secure than 10-20 years ago, even if they do not have formal residence or work permits. One could say that migrant women as caregivers have become part and parcel of Italian family culture. This conclusion is, however, not so much based on recent published research (an exception is Marchetti, 2015) but more on casual observations and informal conversations with Italian academics, social workers and citizens in general. The normalisation of *badanti* does not imply, however, that families fully trust them and welcome them as quasi-relatives. Suspicions, as described in this paper, often continue to make the contract uneasy and fragile.

This study reveals two important lacunae in our knowledge and understanding of migrant care of elderly people in Italy. As we have pointed out, *badanti* were often reluctant to be interviewed, but the older people in their care were even more difficult to approach and were thus completely absent as interlocutors in this paper. Their negative as well as positive appreciation of the care they received was reported to us by others, mostly relatives and *badanti*. Future research is needed to ascertain with more confidence our final conclusion in the last paragraph of this paper. A second suggestion for further study concerns the recent so-called refugee crisis: has the influx of refugees and other migrants in the past three years affected the conditions of migrant care giving in Italy; and if so, in which ways?

Migration is usually seen as an engine of political and cultural change, of creolisation and hybridisation. Migrants are persons who carry on their backs foreign ideas, foods and fashions, different rules and religions, and new ways of living and dying. Despite persistent attempts to domesticate and integrate newcomers, migrants also force and entice locals to adjust to their presence. In migration, persons, ideas and commodities move together in the continuing creation and recreation of what anthropologists call “culture”.

In the case of the *badanti*, the dominant effect of their appearance on the Italian scene is rather that they facilitate the continuity of old Italian ideals. As Da Roit and Facchini (2010, p. 12) observed, “The function of the *badante* retraces and reproduces the model of informal care that characterised the elderly care given by daughters and daughters-in-law. In a certain way, the fact that the care [provided by Badanti] is the same, although it is offered by others, makes the transition from informal care to commoditised care less traumatic, and perhaps more acceptable”(see footnote 6). Family morals stipulate and glorify the filial care offered by children to their ageing parents in their safe and familiar home surroundings. For the time being, migrants help Italian families to remain Italian by following this tradition, or at least keeping up the appearance of doing so.

## Notes

1. A press release of the Italian Institute for Statistics (ISTAT) on 1 April 2014 stated that 42.3 per cent of people younger than 25 years were unemployed, while 36.4 per cent of people between 15 and 64 years were unemployed.

2. “A central explanatory factor behind the Mediterranean welfare state is a belief that family members have the duty to help each other, and that certain tasks pertaining to the family do not belong to national government” (Calzada and Brooks, 2013, p. 516).

3. Lena Näre (2012, pp. 71-2) was able to meet and observe *badanti* in five Neapolitan households through her personal social network. During her visits, she spent three to six hours in the house chatting with the elderly persons and the caregiver and helping the latter in her work (see also Näre, 2009).

4. Very recently, however, there have been initiatives to migrate older European people to countries where the cost of caring is considerably lower. One such initiative concerns Swiss elderly people living in a care institution in Thailand; see: www.bbc.co.uk/news/health-25438325

5. Romanian and Bulgarian women who are now European citizens are, however, allowed to work legally.

6. Translation by Alice Larotonda.

7. It is not clear, however, how many *badanti* did in fact acquire a residence permit. Various financial, political and other (practical) obstacles prevent migrants from applying for or obtaining such a permit (see http://fieri.it/2009/11/25/la-sanatoria-per-le-badanti-e-le-colf-del-2009-fallimento-o-esaurimento-di-un-modello/). An earlier amnesty offered to undocumented caregivers was given in 2002.

8. It should be noted that the more recent events with refugees fall outside the scope of this study.

## References

[ref001] Aronson J. and Neysmith S.M. (1996), “‘You’re not just in there to do the work’: depersonalizing policies and the exploitation of home care workers’ labor”, Gender & Society, Vol. 10 No. 1, pp. 59-77.

[ref002] Calzada I. and Brooks C. (2013), “The myth of Mediterranean familism”, European Societies, Vol. 15 No. 4, pp. 514-34.

[ref003] Castagnone E., Ester Salis E. and Premazzi V. (2013), Promoting Integration for Migrant Domestic Workers in Italy/International Labour Office, International Migration Programme, International and European Forum of Research on Immigration (FIERI), ILO, Geneva.

[ref004] Chang G. (2000), Disposable Domestics: Immigrant Women Workers in the Global Economy, South End Press, Cambridge, MA.

[ref005] Cheng S.-J.A. (2004), “Contextual politics of difference in transnational care: the rhetoric of Filipina domestics’ employers in Taiwan”, Feminist Review, Vol. 77, pp. 46-64.

[ref006] Christensen K. and Guldvik I. (2013), Migrant Care Workers’ Lived Experiences in the UK and Norway, Social Care Workforce Research Unit, King’s College London, London.

[ref007] Colombo A. and Decimo F. (2009), “Spazi di confidenza. La regolazione della distanza sociale nella collaborazione domestica”, in Cantazaro R. and Colombo A. (Eds), Badanti & Co: Il Lavoro Domestico Straniero in Italia, Il Mulino, Bologna, pp. 253-78.

[ref008] D’Onofrio C. (2011), “Il servizio di cura infermieristica a domicilio: considerazioni e percezioni delle operatrici sanitarie e degli assistiti”, in Pavanello M. and Vasconi E. (Eds), La Promozione della Salute e il Valore del Sangue, Antropologia Medica e Sanità Pubblica, Bulzoni, Roma, pp. 81-97.

[ref009] Da Roit B. (2007), “Changing intergenerational solidarities within families in a Mediterranean welfare state: elderly care in Italy”, Current Sociology, Vol. 55 No. 2, pp. 251-69.

[ref010] Da Roit B. (2010), Strategies of Care: Changing Elderly Care in Italy and the Netherlands, Amsterdam University Press, Amsterdam.

[ref011] Da Roit B. and Facchini C. (2010), Anziani e Badanti. Le Differenti Condizioni di chi è Accudito e di chi Accudisce, Franco Angeli, Milano.

[ref012] Da Roit B. and Naldini M. (2010), “Should I stay or should I go? Combining work and care for an older parent in Italy”, South European Society & Politics, Vol. 15 No. 4, pp. 531-51.

[ref013] Da Roit B. and Sabatinelli S. (2013), “Nothing on the move or just going private? Understanding the freeze on child- and eldercare policies and the development of care markets in Italy”, Social Politics, Vol. 20 No. 3, pp. 430-53.

[ref014] De la Cuesta-Benjumea C. and Roe B. (2014), “The experience of family care-givers and migrant paid care-givers’ relief of burden: a contrasted qualitative analysis”, Ageing & Society, Vol. 34 No. 7, pp. 1219-42.

[ref015] Degiuli F. (2007), “A job with no boundaries: home eldercare in Italy”, European Journal of Women’s Studies, Vol. 14 No. 3, pp. 193-207.

[ref016] Destremau B. (2007), “The dynamics of the globalized migrant domestic labour market in the Mediterranean countries”, unpublished paper, ASSR Mobility Conference, Amsterdam.

[ref017] Fabris L. (2004), “Rapporto di Ricerca. Badanti in Veneto. Emersione e governo del fenomeno”, Sintesi del Rapporto di Ricerca, Gennaio 2003, Regione Veneto, available at: www.meltingpot.it (accessed January 2005).

[ref018] Fedyuk O. (2009), Death in the Life of Ukrainian Labor Migrants in Italy, Migration on Line, Prague, available at: http://migration-research.com/public/docs/uploaded/1388231309.OFedyuk_DeathinthelifeofUkrainianlabormigrantsinItaly_1.pdf (accessed 4 December 2014).

[ref019] Giancristofaro L. (2007), “Badanti straniere e nuove familiarità in Abruzzo”, in Morcellini M., Melotti U. and Agustoni A. (Eds), Mondo Globale e Vita Quotidiana, Infanzia, Adolescenza e Scenari Sociali, Pref. di Eide Spedicato Iengo, Tinari, Chieti, pp. 251-69.

[ref020] Hochschild A.R. (2000), “Global care chains and emotional surplus value”, in Hutton W. and Giddens A. (Eds), On the Edge: Living with Global Capitalism, Jonathan Cape, London, pp. 130-46.

[ref021] INPS (2012), “Osservatorio lavoratori domestici”, available at: www.inps.it/webidentity/banchedatistatistiche/domestici/index01.jsp (accessed 2 April 2014).

[ref022] Karner T.X. (1998), “Professional caring: homecare workers as fictive kin”, Journal of Aging Studies, Vol. 12 No. 1, pp. 69-82.

[ref023] Lutz H. (2008), “Introduction: migrant domestic workers in Europe”, in Lutz H. (Ed.), Migration and Domestic Work. A European Perspective on a Global Theme, Ashgate, Aldershot, pp. 1-10.

[ref024] Lutz H. (2011), The New Maids: Transnational Women and the Care Economy, Zed Books, London.

[ref025] Marchetti A. (2009), “Lavoro e conflitto nel servizio domestico”, in Cantazaro R. and Colombo A. (Eds), Badanti & Co: Il Lavoro Domestico Straniero in Italia, Il Mulino, Bologna, pp. 329-58.

[ref026] Marchetti S. (2013), “Dreaming circularity? Eastern European women and job sharing in paid home care”, Journal of Immigrant & Refugee Studies, Vol. 11 No. 4, pp. 347-63.

[ref027] Marchetti S. (2015), “‘Mum seems happy’: children of dependent people and the difficult task of employing someone who takes care of them”, in Triandafyllidou A. and Marchetti S. (Eds), Employers, Agencies and Immigration: Care Work in Europe, Ashgate, Aldershot, pp. 93-110.

[ref028] Näre L. (2009), “The making of ‘proper’ homes – everyday practices of migrant domestic work in Naples”, Modern Italy, Vol. 14 No. 1, pp. 1-17.

[ref029] Näre L. (2012), “Moral economies of reproductive labour: an ethnography of migrant domestic and care labour in Naples, Italy”, Academic dissertation, University of Helsinki.

[ref030] Parreñas R.S. (2008), “Perpetually foreign: Filipina migrant domestic workers in Rome”, in Lutz H. (Ed.), Migration and Domestic Work. A European Perspective on a Global Theme, Ashgate, Aldershot, pp. 99-112.

[ref100] Parreñas R. (2012), “Global care, local configurations – challenges to conceptualizations of care”, Global Networks, Vol. 12 No. 2, pp. 155-74.

[ref031] Piercy K.W. (2000), “When it is more than a job: close relationships between home health aides and older clients”, Journal of Aging & Health, Vol. 12 No. 3, pp. 362-87. 11067702 10.1177/089826430001200305

[ref101] Pollastri C. and Tozzi M. (1999), “Le potenzialità di sviluppo dei mercati di qualità sociale”, in De Vincenti C. and Gabriele S. (Eds), I Mercati di Qualità Sociale. Vecchi e Nuovi Modelli di Consumo, Laterza, Rome and Bari, pp. 79-115.

[ref032] Polverini F. and Lamura G. (2004), Labour Supply in Care Services: National Report on Italy, European Foundation for the Improvement of Living and Working Conditions, INRCA, Ancona.

[ref048] Razavi S. and Staab S. (2010), “Underpaid and overworked – a cross-national perspective on care workers”, International Labour Review, Vol. 149 No. 4, pp. 407-22.

[ref034] Rugolotto S. (2005), “Beyond family reciprocity. The phenomenon of the badanti: immigrant women as non-kin caregivers for frail elderly in Veron”, unpublished master thesis, Medical Anthropology, University of Amsterdam.

[ref035] Scrinzi F. (2008), “Migrations and the restructuring of the welfare state in Italy: change and continuity in the domestic work sector”, in Lutz H. (Ed.), Migration and Domestic Work. A European Perspective on a Global Theme, Ashgate, Aldershot, pp. 29-42.

[ref036] Slany K., Kontos M. and Liapi M. (Eds) (2010), Women in New Migrations: Current Debates in European Societies, Jagiellonian University Press, Cracow.

[ref037] Triandafyllidou A. and Marchetti S. (2013), “Migrant domestic and care workers in Europe: new patterns of circulation?”, Journal of Immigrant & Refugee Studies, Vol. 11 No. 4, pp. 339-46.

[ref038] Triandafyllidou A. and Marchetti S. (2015), “Introduction: the employers’ perspective on migrant domestic and care work”, in Triandafyllidou A. and Marchetti S. (Eds), Employers, Agencies and Immigration: Paying for Care, Ashgate, Aldershot, pp. 1-16.

[ref039] Ungerson C. (2004), “Whose empowerment and independence? A cross-national perspective on ‘cash for care’ schemes”, Ageing & Society, Vol. 24 No. 2, pp. 189-212.

[ref040] Van der Geest S., Mul A. and Vermeulen H. (2004), “Linkages between migration and the care of frail older people: observations from Greece, Ghana and The Netherlands”, Ageing & Society, Vol. 24 No. 3, pp. 431-50.

[ref041] Walton-Roberts M. (2012), “Contextualizing the global nurse care chain: international migration and the status of nursing in Kerala, India”, Global Networks, Vol. 12 No. 2, pp. 175-94.

[ref042] Yeates N. (2012), “Global care chains: a state-of-the-art review and future directions in care transnationalization research”, Global Networks, Vol. 12 No. 2, pp. 135-54.

